# Aortoesophageal Fistula after Endovascular Aortic Aneurysm Repair of a Mycotic Thoracic Aneurysm

**DOI:** 10.1155/2011/649592

**Published:** 2011-09-06

**Authors:** Elizabeth Gavens, Zehra Zaidi, Wissam Al-Jundi, Palepu Kumar

**Affiliations:** Department of Vascular Surgery, Chesterfield Royal Hospital NHS Foundation Trust, Calow, Chesterfield, Derbyshire S44 5BL, UK

## Abstract

Mycotic aneurysms constitute a small proportion of aortic aneurysms. Endovascular repair of mycotic aneurysms has been applied with good short-term and midterm results. However, the uncommon aortoenteric fistula formation remains a potentially fatal complication when repairing such infective aneurysms. We present the case of an 80-year-old woman with thoracic and abdominal aortic mycotic aneurysms, which were successfully treated with endografting. However, the patient presented 3 months later with upper gastrointestinal bleeding secondary to erosion of the thoracic graft into the oesophagus. The patient was treated conservatively due to the high risk of surgical repair. There is currently little exposure to the management of mycotic aortic aneurysms. If suspected, imaging of the entire vasculature will aid initial diagnosis and highlight the extent of the disease process, allowing for efficient management. Aortic endografting for mycotic thoracic aneurysms is a high-risk procedure yet is still an appropriate intervention. Aortoenteric fistulae pose a rare but severe complication of aortic endografting in this setting.

## 1. Introduction

Almost all clinicians at some point in their career will have come across a patient with an aortic aneurysm. However, infectious or mycotic aneurysms only account for a mere 1–3% of all aortic aneurysms [1]. Their nonspecific presentation of fever, malaise, and leukocytosis, poses a particular challenge to diagnosis and management, which is further compounded by the underlying infectious process [2].

The gold standard management strategy remains surgical resection and debridement of the infected aorta and the surrounding tissues, the use of muscle flaps or omentum to cover the infected field and either an interposition graft or extra-anatomical bypass can be followed by long-term antibiotic therapy [3]. However, such surgical management in these patients possesses high surgical risk and mortality [3]. Moreover, most patients with mycotic aneurysms have high significant comorbidities, resulting in a lethal risk from major surgery. With the increased application of aortic endografting, there is an increasing confidence for its use in the treatment of both degenerative and mycotic aneurysms. However, there is limited evidence investigating the effectiveness of aortic endografting in managing mycotic aneurysms due to scarcity of this disease [2].

Secondary aortoenteric fistula (AEF) is a rare but well-known complication after open abdominal aortic aneurysm (AAA) repair, occurring in 0.4–1.6% of cases [4]. It has been also rarely reported following aortic endografting. Chenu et al. [5] have reviewed the 16 reported cases of aortoenteric fistulae after aortic endografting; infection and local inflammation were found to be the most acceptable mechanism underlying fistula formation. The review concluded that AEF postaortic endografting is difficult to diagnose and treat. 

We describe the case of a patient with an aortooesophageal fistula secondary to erosion of endovascular stent deployed for a mycotic thoracic aneurysm.

## 2. Case Report

An 80-year-old lady presented to the emergency department with haematemesis, melaena, and back pain of 1-day duration. Previously, she had undergone aortic endografting procedures for an abdominal and a thoracic mycotic aortic aneurysm (4 months and 3 months, resp.). Her past medical history included stage A chronic lymphoblastic leukaemia (CLL) diagnosed 12 years ago which was under surveillance with no current active treatment. 

**Figure 1:**
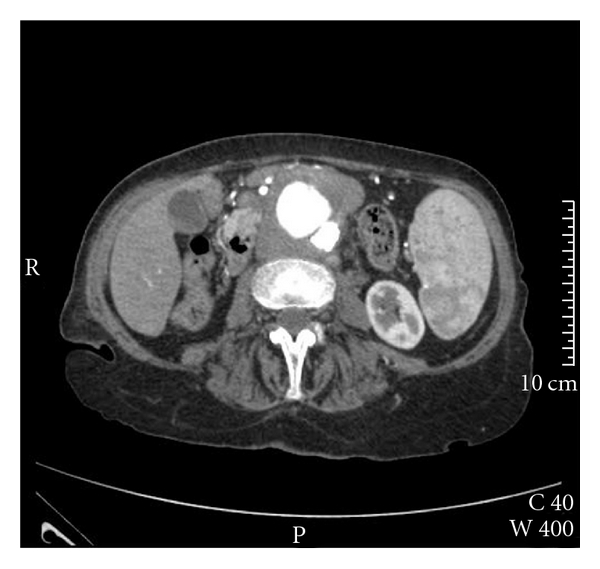
CT scan of the abdominal aorta demonstrating a mycotic aneurysm below the renal arteries.

She was referred originally (4 months previously) to the vascular team with persistent abdominal pain and a palpable pulsatile mass. She denied any other symptoms, had not recently lost weight, or had a temperature and urine culture negative on admission. A chest radiograph at that time did not report any abnormality. An abdominal computed tomography (CT) angiogram revealed a saccular AAA, well below the renal arteries, 4 cm in diameter and 3.7 cm in length with inflammatory changes suggestive of periaortitis (Figure 1). The presence of such inflammatory changes as well as high inflammatory markers (white blood cell (WBC) count: 30.2 × 109/L, erythrocyte sedimentation rate (ESR): 120 mm/hr) raised a suspicion of a mycotic abdominal aneurysm. Blood cultures were obtained but did not grow any microorganisms. It was decided the patient was not fit enough to undergo an open resection and aneurysm repair. After discussion with the patient that curative surgery was likely to prove fatal, she decided to undergo a palliative aortic endografting using Zenith stent-graft (Cook Incorporated, Bloomington, Ind). 

**Figure 2:**
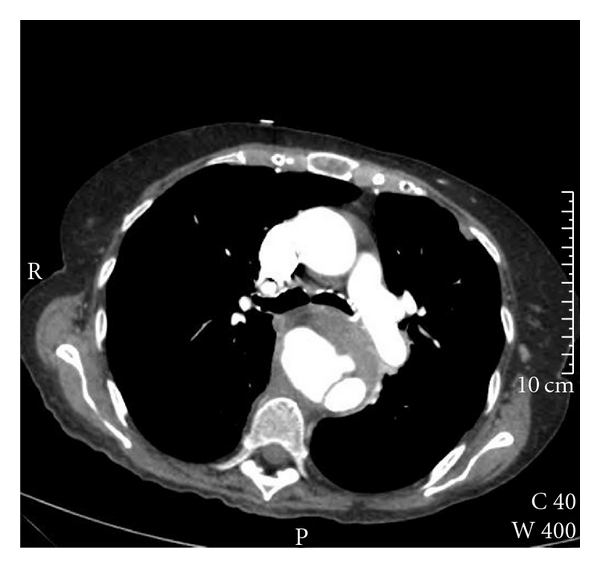
CT scan of the thoracic aorta demonstrating a mycotic aneurysm in the descending thoracic aorta.

Subsequently, she was readmitted 2 weeks later with chest pain, and a chest CT scan revealed another mycotic aneurysm, this time in the descending thoracic aorta. This aneurysm was 6 cm in diameter, 8.5 cm long, and compressing the oesophagus anteriorly (Figure 2). The diagnosis was again discussed with the patient, and, for symptomatic control a further endovascular repair utilising Zenith TX2 stent-graft (Cook Incorporated, Bloomington, Ind) was successfully undertaken and the patient, had an uneventful immediate postoperative recovery. 

She was put on long-term antibiotics to prevent recurrent septicaemia. Investigations were carried out for other septic foci, including an echocardiogram; however, nothing was demonstrated and blood cultures always remained negative. 

Her last surveillance CT angiogram was 3 months after her original admission and demonstrated both stents to be well positioned without any evidence of extraluminal leak. 

**Figure 3:**
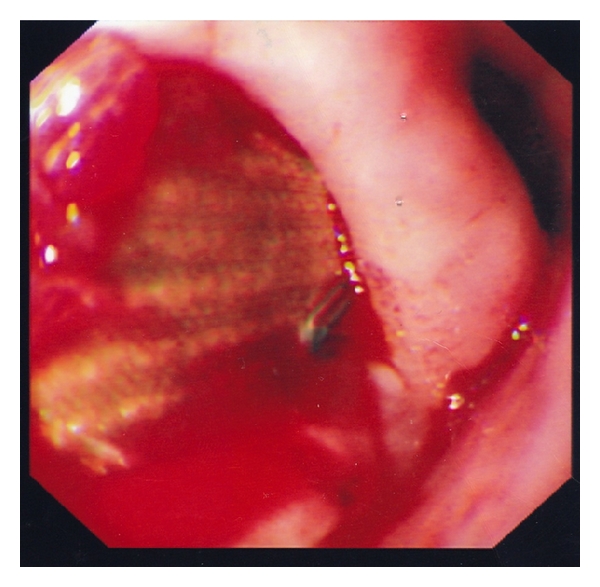
Esophagogastroscopy showing bleeding in the mid oesophagus with the aortic stent eroding into the oesophagus.

At the time of current admission (4 months after she initially presented), the patient had haematemesis, meleana, and back pain. Initial investigations showed haemoglobin of 6.5 g/dL, WBC count of 13.8 × 109/L (neutrophils 4.6 × 109/L, lymphocytes 8.7 × 109/L), Urea 6.8 mmol/L, and creatinine 70 umol/L. Emergency esophagogastroscopy revealed erosion of the thoracic aortic stent into the oesophagus with active bleeding (Figure 3). The bleeding points were injected with adrenaline with good effect.

An urgent multidisciplinary team meeting including a consultant vascular surgeon, consultant vascular radiologist, consultant gastroenterologist, and consultant haematologist agreed that any further operative treatment was likely to prove fatal. The patient and her family were informed of this advice, and, after further discussion, the decision was made to proceed with active supportive medical treatment, including intravenous omeprazole and blood transfusion, but no further surgical procedures were contemplated. 

The lady was transferred to hospice care on analgesia, and regular antibiotics and she died 33 days later due to general deterioration and sepsis.

## 3. Discussion

Mycotic aortic aneurysms account for only 1–3% [1] of all aortic aneurysms and can occur in previously normal aorta although the presence of an aneurysm does predispose to a mycotic aneurysm [6]. Any arterial lesion, including infective endocarditis, can also be considered a causative factor of a mycotic aneurysm. Risk factors, rather than direct causative elements, include immunosuppression, intravenous drug use, and septicaemia [7]. The relative rarity and preference for the thoracic portion of the aorta of mycotic aneurysms increases the complexity of their management [2]. This is further complicated by the fact that the first presentation is often with aneurysmal rupture, which is unfortunately associated with lethal consequences [2, 6]. 

In this case report, the initial radiological investigation was a CT angiogram of the abdominal aorta, which diagnosed the infrarenal mycotic aneurysm. A chest radiograph at that time did not report any abnormality. However, within a few weeks, she represented with symptoms warranting a CT thorax, and subsequently the thoracic aneurysm was diagnosed. It is unclear as to whether she had developed this pathology in her postoperative phase, or whether it was indeed present prior to/at the same time as the abdominal aortic aneurysm. It has been shown that mycotic aneurysms are often multiple and have a predilection to affect the thoracic aorta and the visceral segments [2]. Hence, we propose that one should consider scanning the entire vascular system whenever a mycotic aneurysm is diagnosed to exclude further infected segments. 

The “classic” presentation of AEF involves Chiari's triad of aortoesophageal syndrome—chest pain, episode of small haematemesis followed by massive Haematemesis [6]. AEF is a severe, life-threatening condition with a high morbidity and mortality [8]. The definitive management of this condition is open surgical correction of the fistula and oesophageal repair. However, in some cases, thoracic endovascular aortic aneurysm repair has been shown to serve as a bridge to surgery, allowing patient optimisation [8–10].

Aortic endografting is now a well-established mode of treatment for aortic aneurysms. It has been used in many cases of mycotic aneurysm with mixed results. The concept of deploying a prosthetic graft into the site of infection is indeed controversial [1–3, 5]. Clough et al. [2] found that aortic endografting for mycotic aneurysm is a feasible procedure, with concomitant antibiotic therapy crucial to the management. However, despite antibiotic use, there were still concerns over ongoing infection, as demonstrated with this case. 

Like any intervention, aortic endografting does not come without complications, most commonly postoperative infection, endoleak, graft migration, aortic rupture, aortic thrombosis, and endotension. AEF as a complication of thoracic aortic endografting is rare and has an extremely poor prognosis. There are many aetiologies, leading to the formation of such fistulae, the most common being aortic wall erosion by the stent. 

This is postulated to be due to progressive erosion through the oesophageal and aortic walls by the rigid extremities of the stent itself [5, 7]. Furthermore, infection and local inflammation alone are common and recognised causative factors for AEF [5]. Thus, the combination of aortic endografting in the light of a mycotic aneurysm may further increase the risk of AEF in these patients [5].

## 4. Conclusion

Mycotic thoracic aneurysms are rare, and there is currently little exposure to their management. If suspected, imaging of the entire aorta will aid initial diagnosis and highlight the extent of the disease process, allowing for efficient management. 

Aortic endografting for mycotic thoracic aneurysms is a high-risk procedure yet is still an appropriate intervention, although not a definitive treatment and should be considered a temporising measure. It allows for further discussion with the patient and family, the option to further plan open operative resection and repair and provides some symptomatic control. The use of antibiotics concomitantly remains essential to the management of mycotic aneurysms. 

Aortoenteric fistulae pose a rare yet severe complication of aortic endografting, and awareness of this as a potential complication is crucial for early recognition and subsequent management. As this case study shows, a greater than 30-day survival is possible despite severe postoperative complications and following diagnosis of AEF.
